# Identification of hallmarks of lung adenocarcinoma prognosis using whole genome sequencing

**DOI:** 10.18632/oncotarget.5697

**Published:** 2015-10-13

**Authors:** Li Liu, Jiao Huang, Ke Wang, Li Li, Yangkai Li, Jingsong Yuan, Sheng Wei

**Affiliations:** ^1^ Department of Epidemiology and Biostatistics, and the Ministry of Education Key Lab of Environment and Health, School of Public Health, Tongji Medical College, Huazhong University of Science and Technology, Wuhan, PR China; ^2^ Department of Thoracic Surgery, Tongji Hospital, Tongji Medical College, Huazhong University of Science and Technology, Wuhan, PR China; ^3^ Department of Radiation Oncology, Center for Radiological Research, Columbia University Medical Center, New York, NY, USA

**Keywords:** whole genome sequencing, lung adenocarcinoma, overall survival, progression free survival, copy number variation

## Abstract

In conjunction with clinical characteristics, prognostic biomarkers are essential for choosing optimal therapies to lower the mortality of lung adenocarcinoma. Whole genome sequencing (WGS) of 7 cancerous-noncancerous tissue pairs was performed to explore the comparative copy number variations (CNVs) associated with lung adenocarcinoma. The frequencies of top ranked CNVs were verified in an independent set of 114 patients and then the roles of target CNVs in disease prognosis were assessed in 313 patients. The WGS yielded 2604 CNVs. After frequency validation and biological function screening of top 10 CNVs, 9 mutant driver genes from 7 CNVs were further analyzed for an association with survival. Compared with the *PBXIP1* amplified copy number, unamplified carriers had a 0.62-fold (95%CI = 0.43–0.91) decreased risk of death. Compared with an amplified *TERT*, those with an unamplified *TERT* had a 35% reduction (95% CI = 3%–56%) in risk of lung adenocarcinoma progression. Cases with both unamplified *PBXIP1* and *TERT* had a median 34.32-month extension of overall survival and 34.55-month delay in disease progression when compared with both amplified CNVs. This study demonstrates that CNVs of *TERT* and *PBXIP1* have the potential to translate into the clinic and be used to improve outcomes for patients with this fatal disease.

## INTRODUCTION

Lung adenocarcinoma contributes to over 500,000 deaths annually and an average 5-year survival rate of less than 15% despite great advances in cancer therapy [1]. Identifying and characterizing prognostic determinants are essential for aiding in developing better therapeutic strategies to lower its mortality [2]. While clinical and pathological characteristics are considered major determinants in the variability in outcomes of this disease, genetic factors may also contribute [3].

In past decades, molecular biomarkers, especially single nucleotide polymorphisms (SNPs), assessing an individual's genetic predisposition for diseases have shown potential for guiding clinical treatment of lung cancer [4–6]. Recently, increasing evidence suggests that copy number variations (CNVs) may also account for a large proportion of the heritability of lung cancer [7–9].

A CNV is a large structural genetic aberration that consists of duplications or deletions covering more than 1 kb and may result in phenotypic variation through the alteration of biological function or gene expression [10, 11]. Various methods have been developed to detect CNVs at genome-wide and locus-specific levels, including hybridization, polymerase chain reaction (PCR) amplification and fluorescence resonance energy transfer technology (FRET) [12, 13]. Recently, sequencing, especially next generation sequencing technology, has provided us a better tool with which to completely characterize genomic CNVs. Besides, the technology assists in overcoming the hurdle of the unfixed design and imprecise boundaries of these CNVs [14]. Sequencing has presented advance in detection of biomarkers to progression of various cancers. By whole genome sequencing (WGS) or target sequencing, some studies have demonstrated the prognostic prediction role of high copy number alteration burden in the prostate cancer relapse [15], *FGFR1* and *PIK3CA* amplifications in oral cavity squamous cell carcinoma [16], and *MYC* amplification in pancreatic adenosquamous carcinoma and lung adenocarcinomas [17, 18].

Here, we performed comprehensive WGS to detect CNVs associated with carcinogenesis of lung adenocarcinoma. After the assessment of CNV frequency and screening for potential biological function, the most notable CNVs were detected to find the hallmarks of lung adenocarcinoma prognosis in Chinese patients through survival analysis.

## RESULTS

### Participant characteristics

Seven male lung adenocarcinoma patients who were smokers and had no family history of cancer were recruited to participate in WGS. At the time of diagnosis, their age ranged from 47 to 64 years with a mean age of 56 years. Four of the 7 had stage II lung adenocarcinoma, while the rest had stage III. Samples from 114 patients with lung adenocarcinoma that had been diagnosed by histology were used to validate the frequencies of top 10 CNVs by quantitative polymerase chain reaction (qPCR). Among these patients, 69 (60.52%) were male, 59 (51.75%) were smokers, 35 (30.70%) were drinkers and 17 (14.91%) had a family history of cancer. Three hundred thirteen patients with lung adenocarcinoma were recruited to determine if there was an association between target CNVs previously identified and disease prognosis. Among the 313 patients, 180 (57.51%) were male, 158 (50.48%) were smokers, 93 (29.71%) were drinkers, 39 (12.46%) had a family history of cancer, 113 (36.10%) died and 153 (48.88%) presented disease progression during the follow-up period (Table 1). 260 of 313 lung adenocarcinoma patients (83.07%) completed the last follow-up assessment or died during the follow up, with a median survival time of 46.36 months. There were no difference of characteristics between patients lost of follow-up and patients with completed follow-up.

**Table 1:** Characteristics of study population

	Discovery set (*n* = 7)^a^	Validation Set I (*n* = 114)^b^	Validation Set II (*n* = 313)^c^
Age at surgery (mean ± SD)	56.00 ± 5.01	58.96 ± 9.35	57.68 ± 9.49
Sex			
male	7(100.00%)	69(60.52%)	180(57.51%)
female	0(0.00%)	45(39.48%)	133(42.49%)
BMI(mean ± SD)	22.14 ± 4.12	22.04 ± 5.78	23.17 ± 3.19
Smoking Status			
No	0(0.00%)	55(48.25%)	155(49.52%)
Yes	7(100.00%)	59(51.75%)	158(50.48%)
Smoking index^d^			
≤ 20	1(14.29%)	14(24.56%)	42(27.10%)
> 20	6(85.71%)	43(75.44%)	113(72.90%)
Alcohol use			
No	5(71.43%)	79(69.30%)	220(70.29%)
Yes	2(28.57%)	35(30.70%)	93(29.71%)
TNM stage			
I	0(0.00%)	19(17.76%)	78(24.92%)
II	4(57.14%)	20(18.69%)	65(20.77%)
III	3(42.86%)	55(51.40%)	127(40.58%)
IV	0(0.00%)	13(12.15%)	43(13.74%)
Family history of cancer			
No	7(100.00%)	97(85.09%)	274(87.54%)
Yes	0(0.00%)	17(14.91%)	39(12.46%)
Postoperative chemotherapy			
No	4(57.14%)	64(56.14%)	134(42.81%)
Yes	3(42.86%)	50(43.86%)	179(57.19%)
Postoperative radiotherapy			
No	5(71.43%)	84(73.68%)	229(73.16%)
Yes	2(28.57%)	30(26.32%)	84(26.84%)
Recurrence			
No	2(28.57%)	108(94.74%)	286(91.37%)
Yes	5(71.43%)	6(5.26%)	27(8.63%)
Metastasis			
No	4(57.14%)	84(73.68%)	228(72.84%)
Yes	3(42.86%)	30(26.32%)	85(27.16%)
Death			
No	4(57.14%)	59(51.75%)	200(63.90%)
Yes	3(42.86%)	55(48.25%)	113(36.10%)

a Discovery set was used to screen copy number variations correlated with lung adenocarcinoma

b Validation set I was used to verify the frequency of target copy number variations

c Validation set II was used to detect the correlation between target copy number variations and lung adenocarcinoma survival

d Smoking index = cigarette per day × smoking years

### Overview of the somatic CNV landscape

**Figure 1:**
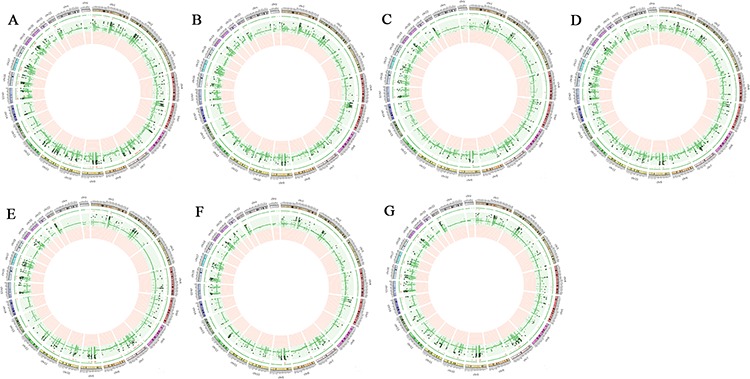
Genomic profiles for comparison between cancerous tissues and paired noncancerous tissues from 7 lung adenocarcinoma patients Green point indicates increased copy number and red point indicates decreased copy number. The circle starts with chromosome 1 and ends with Y chromosome. Only statistical significant amplification and deletion are shown (Fisher's exact test; FDR < 0.05) for each chromosome. **A.** T2-P2 **B.** T4-P4 **C.** T12-P12 **D.** T19-P19 **E.** T25-P25 **F.** T26-P26 **G.** T29-P29

WGS was performed on 7 cancerous and noncancerous paired tissues. The number of reads for a genomic region in the tumor compared to adjacent normal tissue was calculated in order to identify CNVs. The mean sequencing coverage was 2.5× with a range of 2.0× to 5.1 × . 1272, 824 and 2756 CNVs were detected by the CNVseq, BICseq and CNVer algorithm, respectively. After matching CNV areas detected by the three algorithms, 2604 somatic CNVs were identified differently expressed between cancerous and noncancerous tissues, among which, 2488 were amplifications and 116 were deletions. There were 4, 6, 25, 65, 132, 310, 650, 1281 and 131 CNVs were detected with total frequency of 11, 10, 9, 8, 7, 6, 5, 4 and 3, respectively. The distribution of CNVs is presented as Circos plots in Figure 1. The locations of these CNVs on chromosomes are listed in Supplementary Table 1. Chr5_262301_297746, chr5_565873_681306, chr5_1607662_1663720, chr19_37756707_37760335, chr5_209198_257662, chr5_482152_508485, chr5_843417_1023251, chr5_1157300_1368500, chr1_154919397_154921901 and chr3_129101148_129103476 were the top 10 most frequently mutant CNVs detected by the three algorithms (Table 2). The top 100 CNVs and mutant driver genes located in these CNVs are listed in Supplementary Table 2.

**Table 2:** Genes with most significant copy number variant burdens in 7 lung adenocarcinoma patients

CNV Position	Gene Symbol	CNV Status^a^	Total Frequency^b^	Frequency called by BICseq	Frequncy called by CNVseq	Frequency called by CNVer
chr5_262301_297746	*PDCD6*	Amplification	11	5	2	4
chr5_565873_681306	*CEP72*	Amplification	11	5	2	4
chr5_565873_681306	*TPPP*	Amplification	11	5	2	4
chr5_565873_681306	*FREP1*	Amplification	11	5	2	4
chr5_1607662_1663720	*NR_003713*	Amplification	11	5	3	3
chr19_37756707_37760335	*NR_029390*	Amplification	11	4	2	5
chr5_209198_257662	*SDHA*	Amplification	10	5	2	3
chr5_209198_257662	*CCDC127*	Amplification	10	5	2	3
chr5_482152_508485	*SLC9A3*	Amplification	10	5	3	2
chr5_843417_1023251	*NM_001242737*	Amplification	10	5	3	2
chr5_843417_1023251	*BRD9*	Amplification	10	5	3	2
chr5_843417_1023251	*NKD2*	Amplification	10	5	3	2
chr5_843417_1023251	*TRIP13*	Amplification	10	5	3	2
chr5_1157300_1368500	*BC032469*	Amplification	10	5	3	2
chr5_1157300_1368500	*SLC6A19*	Amplification	10	5	3	2
chr5_1157300_1368500	*SLC6A18*	Amplification	10	5	3	2
chr5_1157300_1368500	*TERT*	Amplification	10	5	3	2
chr5_1157300_1368500	*CLPTM1L*	Amplification	10	5	3	2
chr1_154919397_154921901	*PBXIP1*	Amplification	10	4	4	2
chr3_129101148_129103476	*NR_003111*	Amplification	10	3	2	5

a CNV status represents the comparison between cancerous and paired noncarcerous tisses

b Total Frequency represents the sum of CNV frequency detected by three algorithms (BICseq, CNVseq and CNVer)

### Target genotyping of CNVs most frequently detected by qPCR

The biological functions of the single genes located in chr3_129101148_129103476, chr5_1607662_1663720 and chr19_37756707_37760335 are undefined, therefore, only the remaining 7 CNVs of interest were followed-up on. Totally, there were 17 pairs of primers were successfully designed to amplify the 7 CNVs. However, only 12 primers targeting 12 genes presented optimal amplification, which included *PBXIP1*, *SDHA*, *PDCD6*, *SLC9A3*, *CEP72*, *TPPP*, *BRD9*, *TRIP13*, *LOC100506688*, *SLC6A19*, *SLC6A18* and *TERT*. Detailed information on the primers used for the 12 genes is presented in Supplementary Table 3. The rates of amplification of *PBXIP1*, *SDHA*, *PDCD6*, *SLC9A3*, *CEP72*, *TPPP*, *BRD9*, *TRIP13*, *LOC100506688*, *SLC6A19*, *SLC6A18* and *TERT* were 38.68%, 61.61%, 68.18%, 25.44%, 60.98%, 9.65%, 16.38%, 23.68%, 40.35%, 23.48%, 7.89% and 83.78%, respectively (Table 3).

**Table 3:** Frequencies of candidate CNVs in the validation set I by qPCR

Position	Gene Symbol	Frequency^a^	
		Amplification	Nonamplification
chr1_154919397_154921901	*PBXIP1*	41 (38.68%)	65 (61.32%)
chr5_209198_257662	*SDHA*	69 (61.61%)	43 (38.39%)
chr5_262301_297746	*PDCD6*	75 (68.18%)	35 (21.82%)
chr5_482152_508485	*SLC9A3*	29 (25.44%)	85 (74.56%)
chr5_565873_681306	*CEP72*	75 (60.98%)	48 (39.02%)
	*TPPP*	11 (9.65%)	103 (90.35%)
chr5_843417_023251	*BRD9*	19 (16.38%)	97 (83.62%)
	*TRIP13*	27 (23.68%)	87 (76.32%)
	*LOC100506688*	46 (40.35%)	68 (59.65%)
chr5_1157300 _1368500	*SLC6A19*	27 (23.48%)	88 (76.52%)
	*SLC6A18*	9 (7.89%)	105 (92.11%)
	*TERT*	93 (83.78%)	18 (16.22%)

a Patients with different copy numbers were divided into the two groups of amplification and nonamplification, which were distinguished by a cut-off point of 2^−ΔΔCt^ as 1.3.

### CNVs associated with lung adenocarcinoma patient prognosis

CNVs with low amplification (*TPPP* and *SLC6A18*) and undefined biological function (*LOC*100506688) were excluded from further studies on the associations between target CNVs and lung adenocarcinoma prognosis.

**Figure 2:**
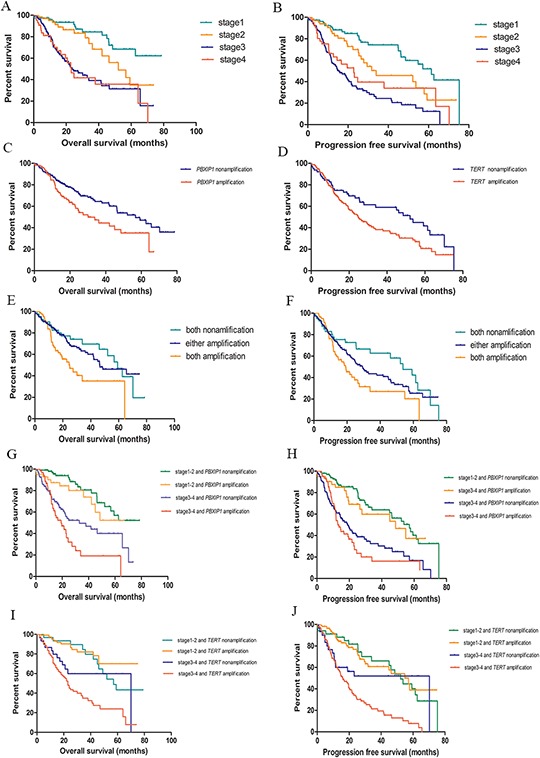
Kaplan-Meier survival curves for survival of lung adenocarcinoma **A.** TNM stage and overall survival; **B.** TNM stage and progression free survival; **C.** *PBXIP1* copy number variations and overall survival of lung adenocarcinoma; **D.** *TERT* copy number variations and progression free survival of lung adenocarcinoma; **E.** Combination of *PBXIP1* and *TERT* copy number variations in overall survival of lung adenocarcinoma; **F.** Combination of *PBXIP1* and *TERT* copy number variations in progression free survival of lung adenocarcinoma; **G.** Combination of clinical stage and *PBXIP1* copy number variations in overall survival of lung adenocarcinoma; **H.** Combination of clinical stage and *PBXIP1* copy number variations in progression free survival of lung adenocarcinoma; **I.** Combination of clinical stage and *TERT* copy number variations in overall survival of lung adenocarcinoma; **J.** Combination of clinical stage and *TERT* copy number variations in progression free survival of lung adenocarcinoma;

From the Cox proportional hazards analysis, significant associations between clinical stage and overall survival and progression free survival were found (Supplementary Table 4). Compared with stage I lung adenocarcinoma patients, stage II, III and IV patients had a 1.79, 4.93 and 6.11-fold increased risk of death, respectively. The corresponding HRs of progression free survival were 1.98, 4.42 and 3.96, respectively (Figure 2A–2B and Supplementary Table 4).

Among 9 genes from the 7 CNV areas of interest, *PBXIP1* was significantly associated with overall survival. Compared with the *PBXIP1* amplified copy number, unamplified carriers had a 0.62-fold (95%CI = 0.43–0.91) decreased risk of death with a prolonged median survival time of 26.63 months. This association remained after adjusting for age, sex, cigarette use, and clinical stage with an HR of 0.58 (95% CI = 0.39–0.86). The 5-year overall survival rate of patients with an unamplified *PBXIP1* was 47.0% (95% CI: 33.3%–56.7%) compared with 35.1% (95% CI: 21.7%–48.8%) in patients with an amplified *PBXIP1* (Table 4 and Figure 2C). Further analysis found that CNV of *TERT* was correlated with progression free survival of lung adenocarcinoma. Compared with an amplified *TERT*, those with an unamplified *TERT* had a 35% reduction (95% CI = 3%–56%) in risk of disease progression. A similar effect was seen after adjusting for age, sex, cigarette use, and clinical staging with an HR of 0.67 (95% CI = 0.45–0.99). In comparison to the amplified variant, the unamplified *TERT* was associated with a median delay in disease progression of 23.77 months. The 3-year progression free survival rate of patients with an unamplified *TERT* was 59.0% (95% CI: 45.0%–70.7%) compared to 38.0% (95% CI: 30.0%–45.9%) of those with an amplified *TERT* (Table 4 and Figure 2D).

**Table 4:** The relationship between copy number variations and prognosis of lung adenocarcinoma

	Overall survival^a^ (*N* = 313)		Progression free survival^b^ (*N* = 313)	
	Crude HR(95%CI)	Adjusted HR^c^(95%CI)	Crude HR(95%CI)	Adjusted HR^c^(95%CI)
*PBXIP1*				
amplification	1.00	1.00	1.00	1.00
nonamplification	0.62(0.43–0.91)	0.58(0.39–0.86)	0.80(0.58–1.11)	0.81(0.58–1.13)
*TERT*				
amplification	1.00	1.00	1.00	1.00
nonamplification	0.81(0.52–1.25)	0.85(0.54–1.32)	0.65(0.44–0.97)	0.67(0.45–0.99)
*CEP72*				
amplification	1.00	1.00	1.00	1.00
nonamplification	0.78(0.53–1.15)	0.88(0.60–1.31)	0.90(0.64–1.25)	1.03(0.73–1.44)
*BRD9*				
amplification	1.00	1.00	1.00	1.00
nonamplification	1.00(0.62–1.62)	1.35(0.82–2.23)	1.20(0.78–1.85)	1.49(0.95–2.33)
*TRIP13*				
amplification	1.00	1.00	1.00	1.00
nonamplification	0.87(0.55–1.38)	1.09(0.68–1.74)	0.99(0.65–1.49)	1.19(0.78–1.81)
*SLC9A3*				
amplification	1.00	1.00	1.00	1.00
nonamplification	1.12(0.66–1.91)	1.07(0.63–1.83	0.88(0.59–1.33)	0.84(0.55–1.27)
*SDHA*				
amplification	1.00	1.00	1.00	1.00
nonamplification	1.15(0.78–1.69)	1.18(0.79–1.75)	0.99(0.71–1.36)	0.95(0.68–1.32)
*SLC6A19*				
amplification	1.00	1.00	1.00	1.00
nonamplification	0.83(0.55–1.25)	0.93(0.61–1.42)	0.95(0.66–1.37)	0.99(0.69–1.44)
*PDCD6*				
amplification	1.00	1.00	1.00	1.00
nonamplification	1.13(0.77–1.66)	1.14(0.77–1.68)	0.83(0.60–1.15)	0.82(0.59–1.15)

a Overall survival was calculated by subtracting the date when the patient was first treated from the date of death, and patients were censored when lost of follow-up.

b Progression free survival was calculated by subtracting the date of first treatment from the date of recurrence of, metastasis of or death from lung adenocarcinoma.

c Adjustment: age, gender, smoking status and TNM stage.

In addition, the combined effect of the CNVs of *PBXIP1* and *TERT* on lung adenocarcinoma prognosis was assessed. Patients with no amplifications in either gene had a 34.32-month longer median survival time with an HR of 0.51(95% CI = 0.28–0.93) compared with those with both genes amplified. Similarly, cases with both unamplified genes had a 34.55-month delay in disease progression compared with patients with amplifications in both genes (HR = 0.55, 95% CI = 0.32–0.94). Even patients with only one nonamplification had a 11.63-month longer overall survival compared with those with both amplifications (HR = 0.54, 95% CI = 0.35–0.84) (Table 5 and Figure 2E–2F).

**Table 5:** The interactions between copy number variations and TNM stage on the prognosis of lung adenocarcinoma

	Overall survival^a^ (*N* = 313)		Progression free survival ^b^(*N* = 313)	
	Crude HR(95%CI)	Adjusted HR(95%CI)	Crude HR(95%CI)	Adjusted HR(95%CI)
*PBXIP1* and *TERT*				
both amplification	1.00	1.00	1.00	1.00
either amplification	0.53(0.35–0.81)	0.54(0.35–0.84)^c^	0.72(0.50–1.03)	0.76(0.52–1.10)^c^
both nonamplification	0.52(0.29–0.94)	0.51(0.28–0.93)^c^	0.52(0.31–0.89)	0.55(0.32–0.94)^c^
Stage and *PBXIP1*				
stage 3–4 and amplification	1.00	1.00	1.00	1.00
stage 3–4 and nonamplification	0.64(0.41–1.00)	0.59(0.37–0.93)^d^	0.89(0.60–1.31)	0.85(0.57–1.27)^d^
stage 1–2 and amplification	0.28(0.14–0.55)	0.26(0.13–0.53)^d^	0.37(0.21–0.66)	0.36(0.20–0.66)^d^
stage 1–2 and nonamplification	0.18(0.10–0.33)	0.15(0.08–0.28)^d^	0.29(0.18–0.47)	0.27(0.17–0.44)^d^
Stage and *TERT*				
stage 3–4 and amplification	1.00	1.00	1.00	1.00
stage 3–4 and nonamplification	0.53(0.28–0.98)	0.50(0.27–0.94)^d^	0.48(0.28–0.84)	0.46(0.26–0.80)^d^
stage 1–2 and amplification	0.19(0.11–0.33)	0.18(0.10–0.32)^d^	0.28(0.19–0.43)	0.28(0.19–0.42)^d^
stage 1–2 and nonamplification	0.38(0.21–0.68)	0.35(0.19–0.62)^d^	0.32(0.19–0.53)	0.30(0.18–0.52)^d^

a Overall survival was calculated by subtracting the date when the patient was first treated from the date of death, and patients were censored when lost of follow-up.

b Progression free survival was calculated by subtracting the date of first treatment from the date of recurrence of, metastasis of or death from lung adenocarcinoma.

c Adjustment: age, gender, smoking status and TNM stage.

d Adjustment: age, gender and smoking status.

We also explored the effect of the two genes in CNVs and clinical stage on lung adenocarcinoma prognosis. Compared with stage III-IV patients with an amplified *PBXIP1*, stage I-II patients with an unamplified *PXBIP1* had an 85% lower risk of death (95% CI = 72%–92%) and a 73% lower risk of progression (95% CI = 56%–83%). Similarly, stage I-II patients with an unamplified *TERT* had a 65% lower risk (95% CI = 38%–81%) of death than stage III-IV patients with an amplified *TERT* and a 70% lower risk (95% CI = 48%-82%) of disease progression (Table 5 and Figure 2G–2J).

The genes *CEP72, BRD9, TRIP13, SLC9A3, SDHA, SLC6A19* and *PDCD6* did not have any predictive role in lung adenocarcinoma prognosis (Table 4). There was no association between the 9 mutant driver genes and the prognosis of patients with lung squamous cell carcinoma (Supplementary Table 5), which supports the concept that lung adenocarcinoma and lung squamous cell carcinoma are genetically heterogeneous cancer types.

### CNVs and expression of *PBXIP1* and *TERT* based on TCGA

**Figure 3:**
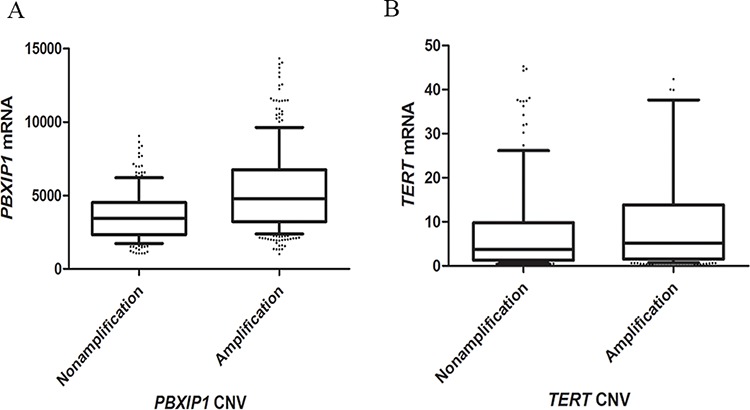
Box plot of gene expression according to copy number variants of *PBXIP1* **A.** and *TERT* **B.** Upper horizontal line of box, 75th percentile; lower horizontal line of box, 25th percentile; horizontal bar within box, median; upper horizontal bar outside box, 90th percentile; lower horizontal bar outside box, 10th percentile. The median gene expressions for the unamplified and amplified *PBXIP1,* and unamplified and amplified *TERT* were 3448.71, 4764.52, 3.77 and 5.16 for, respectively.

From The Cancer Genome Atlas (TCGA) database, whole genome copy number variations of 511 lung adenocarcinoma and whole gene expressions of 512 lung adenocarcinoma were downloaded. Based on the data from TCGA, the amplification rates of *PBXIP1* and *TERT* were 58.8% and 64.1%. Along with an increased copy number, expression of *PBXIP1* and *TERT* mRNA increased significantly. The median gene expressions for unamplified and amplified *PBXIP1* were 3448.71 and 4764.52, respectively (*P* < 0.001). Similarly, the median gene expressions for unamplified and amplified *TERT* were 3.77 and 5.16, respectively (*P* = 0.041). (Figure 3)

## DISCUSSION

To our knowledge, this is the first study on the association between CNVs and the prognosis of lung adenocarcinoma in Chinese patients using WGS. Overall, we found a gain in the short arm of chromosome 5 (5p) correlated with lung adenocarcinoma carcinogenesis. Also, the CNVs of *PBXIP1* and *TERT* presented significant upregulation of corresponding gene expressions and were found to be independently predictive of lung adenocarcinoma patient survival. Furthermore, these two structural mutations strengthen the clinical role of stage in disease prognosis.

Deep sequencing is a well-known technology that has resulted in the most comprehensive collection of biomarkers for various diseases, including cancers [19]. The development of sequencing technologies has opened the door to novel methods for detecting genetic mutations using low-coverage sequencing. Recent published studies have demonstrated the comparability of low-coverage sequencing to deep sequencing for detecting large structural mutations, especially for mutations larger than 20 kb [20]. Additionally, three CNV calling algorithms, CNVseq, CNVer and BICseq, were incorporated into the analysis to improve upon statistical power. CNVseq and BICseq captured many more CNVs than CNVer, and BICseq had a higher detection rate than CNVseq for the same CNVs. Assessment of CNVs using three algorithms may reduce the false positives and negatives normally obtained when using a single algorithm.

In this study, a significant role for the short arm of chromosome 5 (5p) was found in carcinogenesis. Previous molecular cytogenetic studies have shown that chromosomal aberrations occur on 5p in all major lung tumor types [21–25]. Besides a role in carcinogenesis, aberrations in 5p are also biomarkers for cancer prognosis. A genome-wide analysis revealed that copy number gains in chromosome 5p were correlated with to the survival of early-stage non-small cell lung cancer [26]. Sarit *et al*. even presented direct evidence for this with the finding that amplification of genes in chromosome 5p may be responsible for the malignant progression of bronchioloalveolar carcinoma, which is a subtype of lung adenocarcinoma [27].

Telomerase is an enzyme consisting of a reverse transcriptase called telomerase reverse transcriptase (*TERT*) and an RNA component that adds repeats of a DNA sequence (TTAGGG) to the ends of chromosomes in order to prevent shortening. Telomerase activity is high in embryonic and stem cells but nearly undetectable in most somatic cells, due primarily to transcriptional downregulation of *TERT*. However, recent identification of highly recurrent point mutations in the *TERT* promoter in multiple cancer types suggests one potential mechanism for up-regulation of telomerase via reactivation of *TERT* [21, 28]. The importance of *TERT* reactivation in cellular immortalization and carcinogenesis is supported by its expression in more than 90% of immortal cell lines and tumors. The gain of *TERT* is the most frequent amplification event occurring in early stage cancers [29]. A recently published whole genome study directly supports our finding by demonstrating amplification of *TERT* in lung adenocarcinoma [27]. Additionally, overexpression of *TERT* is a biomarker for the progression of and poor outcomes from lung cancer [30–33].

Pre-B-cell leukemia homeobox (PBX) interacting protein 1 (PBXIP1) is a scaffolding protein of the PBX-family interacting microtubule-binding protein. It promotes cell migration which is necessary for cancer cell proliferation, migration and invasion through activation of the PI3K/AKT/mTOR and Raf/MEK/ERK pathways [34]. Little direct evidence has been published for the carcinogenic role of *PBXIP1* in lung adenocarcinoma. However, a recent study indicated that the gene was overexpressed in breast infiltrative ductal carcinoma, as well as promoted cell adhesion and migration through modulation of focal adhesion dynamics. Similar overexpression of *PBXIP1* was also found in high-grade glioma and ependymoma [35], oral squamous cell carcinoma [36] and liver cancer [37]. Moreover, an amplified copy number of *PBXIP1* was found to be predictive of poor outcomes in undifferentiated pleomorphic sarcomas and leiomyosarcomas [38]. Overall, the above evidence supports plausibly role for *PBXIP1* in promoting lung adenocarcinoma.

We acknowledge several limitations to our study. First, the modest sample size of the WGS may not have had optimal statistical power to identify and validate some well-known lung cancer-related genes, such as *TP53*, *EGRF*, *KRAS* and *BRAF*. In our study, these genes were captured by the low-coverage sequencing with relative low frequencies of 19.0%, 28.6%, 28.6% and 9.5%, which was not significant enough to follow-up on in further association studies. Second, given the small sample size in WGS, we picked up 7 typical lung adenocarcinoma patients with similar histology to scan potential somatic copy number variations associated with the disease. Further validation of the positive findings was conducted in general lung adenocarcinoma patients to ensure the good extrapolation of final results. Since the characteristics of the discovery set and validation sets were not consistent especially in gender and smoking status, CNVs contributing to female or non-smoking lung adenocarcinoma may be underestimated. Third, although three copy number calling algorithms were used during analysis, low-coverage sequencing is not as sensitive and specific as deep-sequencing at detecting small structural mutations. This may explain why most of the target CNVs detected were larger than 20 kb. Fourth, we only selected top 7 frequently detected CNVs in the discovery set for further validation. The selection may omit some important CNVs with lower mutation frequency.

In conclusion, this study advances the complete characterization of the genomic CNVs in lung adenocarcinoma in Chinese patients and expands our understanding of tumor biology. Furthermore, a prognostic significance for the CNVs of *TERT* and *PBXIP1* in lung adenocarcinoma was found, which may lead to translation into the clinic and improve outcomes for patients with this fatal disease.

## MATERIALS AND METHODS

### Ethics statement

This study protocol was reviewed and approved by the Institutional Review Board of Huazhong University of Science and Technology. All patients in this study gave written informed consent. This study was carried out in accordance with the recommendations of the Declaration of Helsinki for biomedical research involving human subjects.

### Study population

This study included three populations (discovery set, validation set I and validation set II). Discovery set was used to scan the differently expressed copy number variations between cancerous and paired noncancerous tissues by WGS. Validation set I was used to verify the frequency of top CNVs found in the discovery set. Validation set II was applied to detect the correlation between mutant driver genes from target CNVs with prognosis of lung adenocarcinoma. The population of discovery set and validation sets were from the same library of lung adenocarcinoma patients, but recruited in different stages. The participants of discovery set and validation set I were recruited from Tongji Hospital, Wuhan, China in 2007. The participants of validation set II were recruited from the same hospital since 2008 up to 2013. Follow-up was conducted by researchers since three months later after surgical resection of the tumor from April 2008 to December 2014. Patients with indefinable histological type or lost of follow-up at the first time were excluded from this study. Totally, 434 lung adenocarcinoma patients were recruited. Besides, 303 lung squamous cell carcinoma patients were identified from 2008 to 2013. Questionnaires were used to collect information on patient demographics and lifestyles, including concerning age, gender, cigarette use, alcohol use, family history of cancer, and body mass index (BMI). Participants who had smoked ≥100 cigarettes in their lifetime were defined as “ever smokers”, while those who had smoked fewer were classified as “never smokers”. Similarly, participants who consumed alcoholic beverages at least once a week for ≥1 year were defined as “ever drinkers”, while the remaining cases were “never drinkers”. Patients with any first and/or second-degree relative(s) with a history of cancer were defined as “with a family history of cancer”, while the remaining subjects were “without a family history of cancer”. Patient's clinical data were obtained from medical records. Tumors were staged according to the Union for International Cancer Control (UICC) tumor-node-metastasis (TNM) staging system. The primary endpoint of follow-up was overall survival and the secondary outcome was progression free survival. Overall survival was calculated by subtracting the date when the patient was first treated from the date of death, and patients were censored when lost of follow-up. Progression free survival was calculated by subtracting the date of first treatment from the date of recurrence of, metastasis of or death from lung adenocarcinoma. Patients were censored if death was due to other causes or the annual follow-up was unsuccessful.

### Detection of CNVs by WGS

Paired cancerous and noncancerous tissues from 7 typical lung adenocarcinoma patients with similar histology types recruited in 2007 were enrolled into WGS. Using haematoxylin and eosin (H&E) staining, cancerous tissues were identified as areas made up of more than 80% tumor cells, while noncancerous tissues were defined as areas lacking tumor cells. DNA extraction was then performed using the TIANGEN DNA kit (TIANGEN BIOTECH, Beijing, China) according to the manufacturer's instructions. Sequence capture, enrichment and elution from 14 genomic DNA (gDNA) samples were performed by IntegraGen using Agilent in-solution enrichment (SureSelect Human All Exon Kit v2) with the provided biotinylated oligonucleotide probe library (Human All Exon v2–46 Mb). Briefly, 3 μg of each gDNA sample were sonicated and purified to yield fragments of 150–200 bp. Adaptor oligonucleotides were ligated onto A-tailed fragments and enriched for using 4–6 PCR cycles. The purified libraries, 500ng/library, were hybridized to the SureSelect library for 24 h. Then the eluted fraction was PCR amplified for 10–12 cycles and sequenced on an Illumina HiSeq2000 sequencer as paired-end 75-bp reads [39]. Image analysis and base calling were performed using the Illumina Real Time Analysis (RTA) Pipeline version 1.9 with default parameters. Initial analysis of WGS was based on the Illumina pipeline (CASAVA1.7) against the reference genome of hg19. Because none of the algorithms were optimal for the detection of CNV and to improve the power and compensate for the disadvantage of using a single algorithm, three algorithms, CNVseq [40], CNVer [41] and BICseq [42], were used to identify CNVs in each tumor against the matched noncancerous tissues. The packages used for CNVseq, CNVer and BICseq were CNV-seq, BIC-seq2.1.1 and CNVer-0.81, respectively. CNVs called by each algorithm were produced with the corresponding frequency among 7 patients and then matched with each other to find the common parts. The total frequency of each common CNV was summed from the frequencies called by three algorithms. Then CNVs were ranked according to their total frequency. Circos plots were generated for each patient to summarize the results from the CNV analyses [43].

### Detection of CNVs by qPCR

To verify the findings from WGS, the frequency of the top CNVs in cancerous tissues from 114 lung adenocarcinoma patients was measured by qPCR. After excluding target genes that had only low levels of amplification or an undefined biological function, verified CNVs were further analyzed for an association with survival in 313 lung adenocarcinomas. To explore whether predictive biomarkers for survival of lung adenocarcinoma may also be applicable to lung squamous cell carcinoma, target CNVs were also measured in 303 lung squamous cell carcinomas by qPCR. Primer Premier 5.0 was used to design primers for each CNV. At least one optimal primer was picked for each target CNV. The primer performance was confirmed to have an *r*^2^ > 0.99 and an amplification efficiency of 90%–110%. The qPCR reaction was performed in a total of 20 ul containing 10 μl SYBR Green I Master mix (Toyobo, China), 0.8 mM primers and approximately 50 ng of template DNA. The housekeeping gene β-glubin was used as an internal control for normalization. The pooled DNA from peripheral blood lymphocytes from 100 healthy subjects was used as the standard. PCR reactions for each sample were performed in triplicate using a StepOnePlus Real-time PCR System (Applied Biosystem). The raw data were analyzed using StepOne™ Software v2.1. Amplification levels were calculated using the 2^−ΔΔCt^ method, where ^ΔΔ^Ct for a target gene was defined as (^Δ^Ct of lung cancer sample ^−Δ^Ct of standard) and ^Δ^Ct was the difference in threshold cycles for the sample in question normalized against the reference gene of β-glubin. Patients with different copy numbers were divided into the two groups of nonamplification and amplification, which were distinguished by a cut-off point of 1.3.

### CNV and gene expression data from TCGA

To verify the role of CNVs in gene expressions, whole genome copy number alterations (Affymetrix SNP 6.0 SNP array) and mRNA expressions (RNA seq V2 RSEM) of lung adenocarcinoma were downloaded from The Cancer Genome Atlas Project (TCGA) (https://tcga-data.nci.nih.gov/tcga/tcgaHome2.jsp). According to the recommended cut off point, we divided the segment ratio into CNV as follow rule: unamplification was called if the probe log-ratio ≤ 0.18, otherwise, amplification was called as usual recommended.

### Statistical analysis

The Kaplan-Meier curve and log-rank test were used to estimate the differences in overall survival and progression free survival based on individual CNV. The single effect of CNV and combination effects of CNVs and clinical stage on lung adenocarcinoma prognosis were evaluated by Cox proportional hazards model. Furthermore, the Wilcoxon signed-ranks test was used to analyze the association between target CNVs and their gene expression based on data from TCGA. All tests were two sided and with a *P* < 0.05 was considered significant. All statistical analyses were performed using SAS (version 9.4; SAS Institute, Cary, NC).

## SUPPLEMENTARY TABLES


