# Supplementary material for: Identification of hallmarks of lung adenocarcinoma prognosis using whole genome sequencing
Source: Oncotarget. 2015 Oct 13;6(35):38016–28. doi: 10.18632/oncotarget.5697 (PMC4741981; doi:10.18632/oncotarget.5697)
Supplement: Supplementary file 1 [file oncotarget-06-38016-s001.pdf]

SUPPLEMENTARY TABLES

Supplementary Table S1: The distribution of common copy number variations in chromosome incorporating three bioinformatical algorithms

Chromosome	No. of CNV	Proportion (%)
chr1	389	14.94
chr2	65	2.50
chr3	55	2.11
chr4	42	1.61
chr5	219	8.41
chr6	41	1.57
chr7	499	19.16
chr8	51	1.96
chr9	70	2.69
chr10	63	2.42
chr11	103	3.96
chr12	65	2.50
chr13	26	1.00
chr14	22	0.85
chr15	38	1.46
chr16	245	9.41
chr17	262	10.06
chr18	3	0.11
chr19	107	4.11
chr20	27	1.04
chr21	19	0.73
chr22	176	6.76
chrX	9	0.35
chrY	8	0.31

CNV: copy number variation
Three bioinformatical algorithms: BICseq, CNVseq and CNVer

Supplementary Table S2: Genes within the most significant top 100 copy number variants in 7 lung adenocarcinoma patients

CNV Position	Gene Symbol	Total frequency [a]	BICseq	Cnvseq	Cnver
chr5_262301_297746	*PDCD6*	11	5	2	4
chr5_565873_681306	*CEP72*	11	5	2	4
chr5_565873_681306	*FREP1*	11	5	2	4
chr5_1607662_1663720	*NR_003713*	11	5	3	3
chr19_37756707_37760335	*NR_029390*	11	4	2	5
chr5_209198_257662	*SDHA*	10	5	2	3
chr5_209198_257662	*CCDC127*	10	5	2	3
chr5_482152_508485	*SLC9A3*	10	5	3	2
chr5_843417_1023251	*NM_001242737*	10	5	3	2
chr5_843417_1023251	*BRD9*	10	5	3	2
chr5_843417_1023251	*NKD2*	10	5	3	2
chr5_843417_1023251	*TRIP13*	10	5	3	2
chr5_1157300_1368500	*SLC6A19*	10	5	3	2
chr5_1157300_1368500	*SLC6A18*	10	5	3	2
chr5_1157300_1368500	*TERT*	10	5	3	2
chr5_1157300_1368500	*CLPTM1L*	10	5	3	2
chr1_154919397_154921901	*PBXIP1*	10	4	4	2
chr3_129101148_129103476	*NR_003111*	10	3	2	5
chr1_1595251_1599377	*SLC35E2B*	9	3	2	4
chr1_1595251_1599377	*NM_033487*	9	3	2	4
chr1_1595251_1599377	*CDK11A*	9	3	2	4
chr1_228778127_228782501	*NR_002834*	9	4	3	2
chr1_228778127_228782501	*NR_037962*	9	4	3	2
chr10_134892949_135064463	*UTF1*	9	4	3	2
chr10_134892949_135064463	*GPR123*	9	4	3	2
chr10_134892949_135064463	*VENTX*	9	4	3	2
chr10_134892949_135064463	*KNDC1*	9	4	3	2
chr16_2652716_2653962	*NR_015441*	9	3	2	4
chr16_2652716_2653962	*PDPK1*	9	3	2	4
chr16_381659_385105	*AXIN1*	9	3	2	4
chr22_20250119_20251697	*RTN4R*	9	4	3	2
chr3_129104723_129107265	*NR_003111*	9	3	2	4
chr5_1025782_1115400	*NKD2*	9	5	3	1
chr5_1025782_1115400	*SLC12A7*	9	5	3	1
chr5_1371501_1578900	*AYTL2*	9	5	3	1

(*Continued*)

CNV Position	Gene Symbol	Total frequency [a]	BICseq	Cnvseq	Cnver
chr5_1371501_1578900	*SLC6A3*	9	5	3	1
chr5_1371501_1578900	*NR_003263*	9	5	3	1
chr5_1579663_1599343	*NR_003713*	9	5	2	2
chr5_1579663_1599343	*NR_003263*	9	5	2	2
chr5_1600295_1602369	*NR_003713*	9	5	2	2
chr5_303564_304772	*PDCD6*	9	5	2	2
chr5_303564_304772	*AHRR*	9	5	2	2
chr5_359258_481877	*NR_024158*	9	5	3	1
chr5_359258_481877	*C5ORF55*	9	5	3	1
chr5_359258_481877	*EXOC3*	9	5	3	1
chr5_359258_481877	*AHRR*	9	5	3	1
chr5_359258_481877	*SLC9A3*	9	5	3	1
chr5_508772_562298	*SLC9A3*	9	5	3	1
chr5_681997_684577	*FREP1*	9	5	2	2
chr5_684578_686675	*FREP1*	9	5	2	2
chr5_794857_797215	*ZDHHC11*	9	5	2	2
chr5_802068_809537	*ZDHHC11*	9	5	1	3
chr5_811736_815623	*ZDHHC11*	9	5	1	3
chr5_829590_837625	*ZDHHC11*	9	5	2	2
chr5_838120_842711	*ZDHHC11*	9	5	2	2
chr7_102183588_102185066	*POLR2J2*	9	3	2	4
chr7_220926_222103	*FAM20C*	9	5	3	1
chr1_1403870_1407554	*ATAD3C*	8	3	2	3
chr1_1403870_1407554	*ATAD3B*	8	3	2	3
chr1_154890174_154919397	*PMVK*	8	4	4	0
chr1_154890174_154919397	*PBXIP1*	8	4	4	0
chr1_154921901_155065168	*PYGO2*	8	4	4	0
chr1_154921901_155065168	*SHC1*	8	4	4	0
chr1_154921901_155065168	*NR_024163*	8	4	4	0
chr1_154921901_155065168	*CKS1B*	8	4	4	0
chr1_154921901_155065168	*LENEP*	8	4	4	0
chr1_154921901_155065168	*NR_040772*	8	4	4	0
chr1_154921901_155065168	*FLAD1*	8	4	4	0
chr1_154921901_155065168	*DCST2*	8	4	4	0
chr1_154921901_155065168	*ADAM15*	8	4	4	0
chr1_154921901_155065168	*EFNA4*	8	4	4	0
chr1_154921901_155065168	*DCST1*	8	4	4	0

(*Continued*)

CNV Position	Gene Symbol	Total frequency [a]	BICseq	Cnvseq	Cnver
chr1_154921901_155065168	*NR_040773*	8	4	4	0
chr1_154921901_155065168	*NM_001252405*	8	4	4	0
chr1_154921901_155065168	Form 31.pdf	8	4	4	0
chr1_154921901_155065168	*NR_045515*	8	4	4	0
chr1_154921901_155065168	*ZBTB7B*	8	4	4	0
chr1_154921901_155065168	*PBXIP1*	8	4	4	0
chr1_154921901_155065168	*EFNA3*	8	4	4	0
chr10_134068264_134319288	*C10ORF91*	8	3	3	2
chr10_134068264_134319288	*NR_026559*	8	3	3	2
chr10_134068264_134319288	*LRRC27*	8	3	3	2
chr10_134068264_134319288	*STK32C*	8	3	3	2
chr10_134068264_134319288	*PWWP2B*	8	3	3	2
chr10_134335905_134802097	*NKX6–2*	8	3	3	2
chr10_134335905_134802097	*NM_173572*	8	3	3	2
chr10_134335905_134802097	*INPP5A*	8	3	3	2
chr10_134335905_134802097	*C10ORF92*	8	3	3	2
chr11_1266253_1268281	*MUC5B*	8	3	2	3
chr11_72286066_72527667	*ARAP1*	8	4	4	0
chr11_72286066_72527667	*ATG16L2*	8	4	4	0
chr11_72286066_72527667	*STARD10*	8	4	4	0
chr11_72286066_72527667	*PDE2A*	8	4	4	0
chr16_1290398_1293445	*TPSB2*	8	4	2	2
chr16_2168656_2171625	*PKD1*	8	3	2	3
chr16_2184386_2186200	*PKD1*	8	3	1	4
chr16_2214548_2215688	*TRAF7*	8	3	2	3
chr16_2589744_2596702	*PDPK1*	8	3	2	3
chr16_2658404_2664867	*NR_015441*	8	3	2	3
chr16_3102432_3105981	*MMP25*	8	3	2	3
chr16_3119294_3122064	*IL32*	8	3	2	3
chr16_88076990_88121802	*BANP*	8	3	0	5
chr16_88794960_88796150	*PIEZO1*	8	4	2	2
chr16_939886_1277445	*NR_036442*	8	4	3	1
chr16_939886_1277445	*C1QTNF8*	8	4	3	1
chr16_939886_1277445	*TPSG1*	8	4	3	1
chr16_939886_1277445	*SOX8*	8	4	3	1
chr16_939886_1277445	*SSTR5*	8	4	3	1
chr16_939886_1277445	*NR_027242*	8	4	3	1

(*Continued*)

CNV Position	Gene Symbol	Total frequency [a]	BICseq	Cnvseq	Cnver
chr16_939886_1277445	*CACNA1H*	8	4	3	1
chr16_939886_1277445	*NR_033645*	8	4	3	1
chr16_939886_1277445	*NR_033646*	8	4	3	1
chr16_939886_1277445	*LA16C-360B4.1*	8	4	3	1
chr17_43500758_43503582	*ARHGAP27*	8	3	2	3
chr17_43511719_43516864	*PLEKHM1*	8	3	2	3
chr17_43511719_43516864	*NR_027782*	8	3	2	3
chr17_43511719_43516864	*NR_027774*	8	3	2	3
chr17_43527535_43531296	*PLEKHM1*	8	3	2	3
chr17_43527535_43531296	*NR_027782*	8	3	2	3
chr17_43527535_43531296	*NR_027774*	8	3	2	3
chr17_79753350_80300900	*GCGR*	8	4	4	0
chr17_79753350_80300900	*PPP1R27*	8	4	4	0
chr17_79753350_80300900	*ARHGDIA*	8	4	4	0
chr17_79753350_80300900	*ALYREF*	8	4	4	0
chr17_79753350_80300900	*NPB*	8	4	4	0
chr17_79753350_80300900	*PCYT2*	8	4	4	0
chr17_79753350_80300900	*NR_033685*	8	4	4	0
chr17_79753350_80300900	*NR_033683*	8	4	4	0
chr17_79753350_80300900	*NR_033682*	8	4	4	0
chr17_79753350_80300900	*NR_033681*	8	4	4	0
chr17_79753350_80300900	*MAFG*	8	4	4	0
chr17_79753350_80300900	*PYCR1*	8	4	4	0
chr17_79753350_80300900	*NOTUM*	8	4	4	0
chr17_79753350_80300900	*STRA13*	8	4	4	0
chr17_79753350_80300900	*DCXR*	8	4	4	0
chr17_79753350_80300900	*RAC3*	8	4	4	0
chr17_79753350_80300900	*RFNG*	8	4	4	0
chr17_79753350_80300900	*GPS1*	8	4	4	0
chr17_79753350_80300900	*SLC16A3*	8	4	4	0
chr17_79753350_80300900	*CD7*	8	4	4	0
chr17_79753350_80300900	*FAM195B*	8	4	4	0
chr17_79753350_80300900	*P4HB*	8	4	4	0
chr17_79753350_80300900	*ANAPC11*	8	4	4	0
chr17_79753350_80300900	*MYADML2*	8	4	4	0
chr17_79753350_80300900	*NR_015454*	8	4	4	0
chr17_79753350_80300900	*SIRT7*	8	4	4	0

(*Continued*)

CNV Position	Gene Symbol	Total frequency [a]	BICseq	Cnvseq	Cnver
chr17_79753350_80300900	*FASN*	8	4	4	0
chr17_79753350_80300900	*DUS1L*	8	4	4	0
chr17_79753350_80300900	*LRRC45*	8	4	4	0
chr17_79753350_80300900	*SECTM1*	8	4	4	0
chr17_79753350_80300900	*CSNK1D*	8	4	4	0
chr17_79753350_80300900	*CCDC57*	8	4	4	0
chr17_79753350_80300900	*ASPSCR1*	8	4	4	0
chr17_79753350_80300900	*NR_045351*	8	4	4	0
chr19_32859988_34455072	*PDCD5*	8	1	2	5
chr19_32859988_34455072	*RGS9BP*	8	1	2	5
chr19_32859988_34455072	*C19ORF40*	8	1	2	5
chr19_32859988_34455072	*LRP3*	8	1	2	5
chr19_32859988_34455072	*CEBPA*	8	1	2	5
chr19_32859988_34455072	*NR_026887*	8	1	2	5
chr19_32859988_34455072	*ZNF507*	8	1	2	5
chr19_32859988_34455072	*TDRD12*	8	1	2	5
chr19_32859988_34455072	*NUDT19*	8	1	2	5
chr19_32859988_34455072	*SLC7A9*	8	1	2	5
chr19_32859988_34455072	*WDR88*	8	1	2	5
chr19_32859988_34455072	*GPATCH1*	8	1	2	5
chr19_32859988_34455072	*SLC7A10*	8	1	2	5
chr19_32859988_34455072	*CEBPG*	8	1	2	5
chr19_32859988_34455072	*KCTD15*	8	1	2	5
chr19_32859988_34455072	*ANKRD27*	8	1	2	5
chr19_32859988_34455072	*DPY19L3*	8	1	2	5
chr19_32859988_34455072	*CEP89*	8	1	2	5
chr19_32859988_34455072	*CHST8*	8	1	2	5
chr19_32859988_34455072	*PEPD*	8	1	2	5
chr19_32859988_34455072	*RHPN2*	8	1	2	5
chr19_37300087_37753008	*ZNF790*	8	3	2	3
chr19_37300087_37753008	*NR_040028*	8	3	2	3
chr19_37300087_37753008	*NR_040027*	8	3	2	3
chr19_37300087_37753008	*ZNF829*	8	3	2	3
chr19_37300087_37753008	*ZNF568*	8	3	2	3
chr19_37300087_37753008	*ZNF383*	8	3	2	3
chr19_37300087_37753008	*ZNF585A*	8	3	2	3
chr19_37300087_37753008	*ZNF585B*	8	3	2	3

(*Continued*)

CNV Position	Gene Symbol	Total frequency [a]	BICseq	Cnvseq	Cnver
chr19_37300087_37753008	*ZNF345*	8	3	2	3
chr19_37300087_37753008	*NM_001242474*	8	3	2	3
chr19_37300087_37753008	*NR_038362*	8	3	2	3
chr19_37300087_37753008	*ZNF420*	8	3	2	3
chr19_918901_1033690	*WDR18*	8	5	3	0
chr19_918901_1033690	*GRIN3B*	8	5	3	0
chr19_918901_1033690	*C19ORF6*	8	5	3	0
chr19_918901_1033690	*CNN2*	8	5	3	0
chr19_918901_1033690	*ARID3A*	8	5	3	0
chr19_918901_1033690	*KISS1R*	8	5	3	0
chr20_60684801_60948525	*PSMA7*	8	4	3	1
chr20_60684801_60948525	*HRH3*	8	4	3	1
chr20_60684801_60948525	*SS18L1*	8	4	3	1
chr20_60684801_60948525	*GTPBP5*	8	4	3	1
chr20_60684801_60948525	*LSM14B*	8	4	3	1
chr20_60684801_60948525	*ADRM1*	8	4	3	1
chr20_60684801_60948525	*LAMA5*	8	4	3	1
chr20_60684801_60948525	*OSBPL2*	8	4	3	1
chr20_61262394_61311852	*NR_024470*	8	4	4	0
chr20_61262394_61311852	*SLCO4A1*	8	4	4	0
chr20_61935285_61985361	*CHRNA4*	8	4	3	1
chr20_61935285_61985361	*COL20A1*	8	4	3	1
chr22_20251698_20254347	*RTN4R*	8	4	3	1
chr22_20254546_20256977	*RTN4R*	8	4	3	1
chr22_20324921_20339624	*NR_038388*	8	4	1	3
chr22_21779832_21784890	*HIC2*	8	3	2	3
chr3_129111351_129116166	*NR_003111*	8	3	1	4
chr5_1023251_1025782	*NKD2*	8	5	3	0
chr5_134773_209198	*LRRC14B*	8	6	2	0
chr5_134773_209198	*CCDC127*	8	6	2	0
chr5_134773_209198	*PLEKHG4B*	8	6	2	0
chr5_1606081_1607221	*NR_003713*	8	5	2	1
chr5_1715801_1854600	*MRPL36*	8	5	2	1
chr5_1715801_1854600	*NDUFS6*	8	5	2	1
chr5_1858501_1973300	*IRX4*	8	5	2	1
chr5_298699_300087	*PDCD6*	8	5	2	1
chr5_301382_302403	*PDCD6*	8	5	2	1

(*Continued*)

CNV Position	Gene Symbol	Total frequency [a]	BICseq	Cnvseq	Cnver
chr5_357708_358932	*AHRR*	8	5	2	1
chr5_481877_482152	*SLC9A3*	8	5	3	0
chr5_508485_508772	*SLC9A3*	8	5	3	0
chr5_693258_693775	*FREP1*	8	5	2	1
chr5_797216_799821	*ZDHHC11*	8	5	1	2
chr5_800292_801593	*ZDHHC11*	8	5	1	2
chr6_32022076_32025406	*TNXB*	8	3	1	4
chr7_102116199_102117743	*POLR2J1*	8	3	3	2
chr7_102191037_102195679	*SPDYE2L*	8	3	2	3
chr7_102191037_102195679	*SPDYB2-L1*	8	3	2	3
chr7_102191037_102195679	*POLR2J2*	8	3	2	3
chr7_102239099_102243732	*RASA4*	8	3	2	3
chr7_102291859_102294191	*SPDYE2L*	8	3	2	3
chr7_102291859_102294191	*SPDYB2-L1*	8	3	2	3
chr7_102291859_102294191	*POLR2J2*	8	3	2	3
chr7_179294_220926	*FAM20C*	8	5	3	0
chr7_1853099_2030472	*MAD1L1*	8	5	3	0
chr7_2030472_2031591	*MAD1L1*	8	5	1	2
chr7_2074899_2075991	*MAD1L1*	8	5	1	2
chr7_222103_344548	*NM_001195127*	8	5	3	0
chr7_222103_344548	*FAM20C*	8	5	3	0
chr7_44022190_44024191	*NR_003655*	8	4	1	3
chr7_44025114_44026948	*NR_003655*	8	4	1	3
chr7_44027352_44029475	*NR_003655*	8	4	1	3
chr7_44056335_44058955	*NR_003655*	8	4	0	4
chr7_75038778_75047658	*NR_033322*	8	3	1	4
chr7_75038778_75047658	*POM121C*	8	3	1	4
chr7_884098_1180300	*COX19*	8	5	3	0
chr7_884098_1180300	*CYP2W1*	8	5	3	0
chr7_884098_1180300	*UNC84A*	8	5	3	0
chr7_884098_1180300	*ADAP1*	8	5	3	0
chr7_884098_1180300	*GPER*	8	5	3	0
chr7_884098_1180300	*GPR146*	8	5	3	0
chr7_884098_1180300	*GET4*	8	5	3	0
chr7_884098_1180300	*C7ORF50*	8	5	3	0
chr8_144940331_144942304	*EPPK1*	8	4	2	2
chr9_139211354_140633587	*NR_026964*	8	4	4	0

(*Continued*)

CNV Position	Gene Symbol	Total frequency [a]	BICseq	Cnvseq	Cnver
chr9_139211354_140633587	*C9ORF151*	8	4	4	0
chr9_139211354_140633587	*GPSM1*	8	4	4	0
chr9_139211354_140633587	*EGFL7*	8	4	4	0
chr9_139211354_140633587	*NR_024543*	8	4	4	0
chr9_139211354_140633587	*NR_024542*	8	4	4	0
chr9_139211354_140633587	*NR_003672*	8	4	4	0
chr9_139211354_140633587	*LCN10*	8	4	4	0
chr9_139211354_140633587	*LCN8*	8	4	4	0
chr9_139211354_140633587	*TMEM141*	8	4	4	0
chr9_139211354_140633587	*NR_024580*	8	4	4	0
chr9_139211354_140633587	*KIAA1984*	8	4	4	0
chr9_139211354_140633587	*PHPT1*	8	4	4	0
chr9_139211354_140633587	*MAMDC4*	8	4	4	0
chr9_139211354_140633587	*EDF1*	8	4	4	0
chr9_139211354_140633587	*C8G*	8	4	4	0
chr9_139211354_140633587	*LCN12*	8	4	4	0
chr9_139211354_140633587	*LCNL1*	8	4	4	0
chr9_139211354_140633587	*PTGDS*	8	4	4	0
chr9_139211354_140633587	*C9ORF142*	8	4	4	0
chr9_139211354_140633587	*CLIC3*	8	4	4	0
chr9_139211354_140633587	*C9ORF139*	8	4	4	0
chr9_139211354_140633587	*FUT7*	8	4	4	0
chr9_139211354_140633587	*ENTPD2*	8	4	4	0
chr9_139211354_140633587	*SAPCD2*	8	4	4	0
chr9_139211354_140633587	*NR_027447*	8	4	4	0
chr9_139211354_140633587	*UAP1L1*	8	4	4	0
chr9_139211354_140633587	*DPP7*	8	4	4	0
chr9_139211354_140633587	*LRRC26*	8	4	4	0
chr9_139211354_140633587	*ANAPC2*	8	4	4	0
chr9_139211354_140633587	*TPRN*	8	4	4	0
chr9_139211354_140633587	*RNF208*	8	4	4	0
chr9_139211354_140633587	*NDOR1*	8	4	4	0
chr9_139211354_140633587	*SLC34A3*	8	4	4	0
chr9_139211354_140633587	*RNF224*	8	4	4	0
chr9_139211354_140633587	*C9ORF169*	8	4	4	0
chr9_139211354_140633587	*TUBB2C*	8	4	4	0
chr9_139211354_140633587	*NR_038389*	8	4	4	0

(*Continued*)

CNV Position	Gene Symbol	Total frequency [a]	BICseq	Cnvseq	Cnver
chr9_139211354_140633587	*C9ORF173*	8	4	4	0
chr9_139211354_140633587	*FAM166A*	8	4	4	0
chr9_139211354_140633587	*TOR4A*	8	4	4	0
chr9_139211354_140633587	*NRARP*	8	4	4	0
chr9_139211354_140633587	*NOXA1*	8	4	4	0
chr9_139211354_140633587	*MRPL41*	8	4	4	0
chr9_139211354_140633587	*ZMYND19*	8	4	4	0
chr9_139211354_140633587	*C9ORF37*	8	4	4	0
chr9_139211354_140633587	*SDCCAG3*	8	4	4	0
chr9_139211354_140633587	*PMPCA*	8	4	4	0
chr9_139211354_140633587	*CARD9*	8	4	4	0
chr9_139211354_140633587	*SNAPC4*	8	4	4	0
chr9_139211354_140633587	*NOTCH1*	8	4	4	0
chr9_139211354_140633587	*C9ORF163*	8	4	4	0
chr9_139211354_140633587	*SEC16A*	8	4	4	0
chr9_139211354_140633587	*AGPAT2*	8	4	4	0
chr9_139211354_140633587	*NR_045111*	8	4	4	0
chr9_139211354_140633587	*NR_045110*	8	4	4	0
chr9_139211354_140633587	*NR_033913*	8	4	4	0
chr9_139211354_140633587	*LCN6*	8	4	4	0
chr9_139211354_140633587	*LCN15*	8	4	4	0
chr9_139211354_140633587	*FAM69B*	8	4	4	0
chr9_139211354_140633587	*TRAF2*	8	4	4	0
chr9_139211354_140633587	*C9ORF172*	8	4	4	0
chr9_139211354_140633587	*FBXW5*	8	4	4	0
chr9_139211354_140633587	*ABCA2*	8	4	4	0
chr9_139211354_140633587	*NPDC1*	8	4	4	0
chr9_139211354_140633587	*GRIN1*	8	4	4	0
chr9_139211354_140633587	*TMEM203*	8	4	4	0
chr9_139211354_140633587	*SSNA1*	8	4	4	0
chr9_139211354_140633587	*COBRA1*	8	4	4	0
chr9_139211354_140633587	*NELF*	8	4	4	0
chr9_139211354_140633587	*ENTPD8*	8	4	4	0
chr9_139211354_140633587	*WDR85*	8	4	4	0
chr9_139211354_140633587	*INPP5E*	8	4	4	0
chr9_139211354_140633587	*C9ORF86*	8	4	4	0
chr9_139211354_140633587	*PNPLA7*	8	4	4	0

(*Continued*)

CNV Position	Gene Symbol	Total frequency [a]	BICseq	Cnvseq	Cnver
chr9_139211354_140633587	*EXD3*	8	4	4	0
chr9_139211354_140633587	*MAN1B1*	8	4	4	0
chr9_139211354_140633587	*NR_045720*	8	4	4	0
chr9_139211354_140633587	*NR_045721*	8	4	4	0
chr9_139211354_140633587	*EHMT1*	8	4	4	0
chr9_139211354_140633587	*ARRDC1*	8	4	4	0
chr1_1382707_1389750	*ATAD3C*	7	3	1	3
chr1_1460434_1465252	*ATAD3A*	7	3	1	3
chr1_155182101_155185210	*MTX1*	7	4	1	2

[a]Total Frequency represents the sum of CNV frequency detected by three in each algorithms (BICseq, CNVseq and CNVer)

Supplementary Table S3: Optimal primers for candidate genes in qPCR

GeneSymbol	Forward	Reverse
PBXIP1	5′-CCAAACCGCTCAGCCACA-3′	5′-CCGCTCCACTTCCTCCAT-3′
SDHA	5′-TGTGGGTTGCTTTGTATT-3′	5′-AGAAGGTGCCAAGGAGTA-3′
PDCD6	5′-TACAGGATGGGTAAGGTAT-3′	5′-TAAAGACTAAAGCAGGAACT-3′
SLC9A3	5′-CAACATCTCGGAGCAGTCGG-3′	5′-TGAAGACCAGCGTCAGGAGC-3′
CEP72	5′-GTTGAGCCTGACTACCGC-3′	5′-CACAGACACCCACTACGAC-3′
TPPP	5′-GTGGACGAGTCAGGCTATGTG-3′	5′-GGAATGTAATGAAGTGCGAGGT-3′
BRD9	5′-GCCACGACTCCAGTTACTATGA-3′	5′-GCTCCTTTACCTTTCGCTTC-3′
TRIP13	5′-CGCTGTATGTCCAGGTGAG-3′	5′-TGTTTCGGTGCCAAGGTC-3′
LOC100506688	5′-ACACTTATCTTCCAACCTCCTC-3′	5′-CCTCTACCCGACATCCCT-3′
SLC6A19	5′-CAGCAACAACTGCGAGAAGG-3′	5′-CAATGACGGAGTAGACCACGAT-3′
SLC6A18	5′-CAGAAGGATGCGGTGGTC-3′	5′-GTGCTCGTAGTCATTAGTTGCT-3′
TERT	5′-CCTGAGGGCAGAGGTGAT-3′	5′-GATGATGCTGGCGATGAC-3′

Supplementary Table S4: Association between demographical variables, clinical characteristics and prognosis of lung adenocarcinoma

		Lung adenocarcinoma(*N* = 313)	
		Overall survival [a]	Progression free survival [b]
Age	≤60	1.00	1.00
	>60	1.27(0.86–1.89)	1.07(0.76–1.50)
Sex	male	1.00	1.00
	female	0.54(0.31–0.95)	0.64(0.39–1.04)
BMI	<25	1.00	1.00
	≥25	0.93(0.61–1.43)	0.93(0.64–1.34)
Smoking	No	1.00	1.00
	Yes	0.66(0.38–1.16)	0.70(0.43–1.14)
Drinking	No	1.00	1.00
	Yes	1.43(0.85–2.43)	1.25(0.80–1.95)
History of respiratory disease	No	1.00	1.00
	Yes	1.15(0.69–1.93)	1.14(0.74–1.77)
Family history of cancer	No	1.00	1.00
	Yes	0.58(0.31–1.11)	0.71(0.43–1.17)
Stage	I	1.00	1.00
	II	1.79(0.86–3.72)	1.98(1.11–3.53)
	III	4.93(2.71–8.97)	4.42(2.72–7.17)
	IV	6.11(3.02–12.34)	3.96(2.18–7.19)

[a]Overall survival was calculated by subtracting the date when the patient was first treated from the date of death, and patients were censored when lost of follow-up.
[b]Progression free survival was calculated by subtracting the date of first treatment from the date of recurrence of, metastasis of or death from lung adenocarcinoma.
Adjustment: age, gender, smoking status, alcohol use, BMI, history of respiratory disease, family history of cancer and TNM stage.

Supplementary Table S5: The relationship between target copy number variants and prognosis of lung squamonus cell carcinoma

Copy number variation	Overall survival [a] (*N* = 303)		Progression free survival [b] (*N* = 303)	
	Crude HR(95%CI)	Adjusted HR [c] (95%CI)	Crude HR(95%CI)	Adjusted HR [c] (95%CI)
CEP72				
amplification	1.00	1.00	1.00	1.00
nonamplification	0.78(0.53–1.16)	0.71(0.47–1.06)	0.89(0.62–1.29)	0.81(0.56–1.18)
BRD9				
amplification	1.00	1.00	1.00	1.00
nonamplification	0.83(0.50–1.38)	0.77(0.46–1.31)	0.85(0.53–1.37)	0.79(0.48–1.30)
TRIP13				
amplification	1.00	1.00	1.00	1.00
nonamplification	0.89(0.54–1.47)	0.86(0.51–1.45)	0.90(0.56–1.46)	0.87(0.53–1.43)
SLC9A3				
amplification	1.00	1.00	1.00	1.00
nonamplification	1.33(0.75–2.35)	1.09(0.61–1.94)	1.20(0.72–1.99)	1.00(0.60–1.67)
SDHA				
amplification	1.00	1.00	1.00	1.00
nonamplification	1.09(0.71–1.67)	1.12(0.71–1.76)	1.02(0.68–1.53)	1.05(0.68–1.60)
SLC6A19				
amplification	1.00	1.00	1.00	1.00
nonamplification	1.01(0.62–1.62)	1.12(0.66–1.88)	1.10(0.70–1.75)	1.20 (0.73–1.98)
TERT				
amplification	1.00	1.00	1.00	1.00
nonamplification	1.15(0.69–1.89)	1.31(0.79–2.16)	0.99(0.60–1.62)	1.15(0.70–1.89)
PBXIP1				
amplification	1.00	1.00	1.00	1.00
nonamplification	1.12(0.75–1.67)	1.18(0.78–1.78)	1.04(0.72–1.51)	1.09(0.74–1.60)
PDCD6				
amplification	1.00	1.00	1.00	1.00
nonamplification	1.23(0.81–1.85)	1.15(0.75–1.77)	1.07(0.72–1.58)	0.99(0.66–1.49)

[a]Overall survival was calculated by subtracting the date when the patient was first treated from the date of death, and patients were censored when lost of follow-up.
[b]Progression free survival was calculated by subtracting the date of first treatment from the date of recurrence of, metastasis of or death from lung squamonus cell carcinoma.
[c]Adjustment: age, gender, smoking status and TNM stage.
